# Diabetes does not impact the diagnostic performance of contrast-based fractional flow reserve: insights from the CONTRAST study

**DOI:** 10.1186/s12933-016-0494-2

**Published:** 2017-01-13

**Authors:** Giuseppe Gargiulo, Eugenio Stabile, Marco Ferrone, Emanuele Barbato, Frederik M. Zimmermann, Julien Adjedj, Barry Hennigan, Mitsuaki Matsumura, Nils P. Johnson, William F. Fearon, Allen Jeremias, Bruno Trimarco, Giovanni Esposito, On behalf of the CONTRST Study Investigators

**Affiliations:** 1 Department of Advanced Biomedical Sciences, University of Naples Frederico II, Naples, Italy; 2 Cardiovascular Center, OLV Clinic, Aalst, Belgium; 3 Department of Cardiology, Catharina Hospital, Eindhoven, The Netherlands; 4 West of Scotland Heart and Lung Centre, Golden Jubilee National Hospital, Clydebank, Scotland UK; 5 British Heart Foundation Glasgow Cardiovascular Research Centre, Institute of Cardiovascular and Medical Sciences, University of Glasgow, Glasgow, Scotland UK; 6 Cardiovascular Research Foundation (CRF), New York, NY USA; 7 Division of Cardiology, Department of Medicine, Weatherhead PET Center, McGovern Medical School at UTHealth and Memorial Hermann Hospital, Houston, TX USA; 8 Stanford University Medical Center, Stanford, USA; 9 The Palo Alto VA Health Care Systems, Palo Alto, CA USA; 10 Division of Cardiovascular Medicine, Stony Brook University Medical Center, Stony Brook, NY USA; 11 Division of Cardiology-Department of Advanced Biomedical Sciences, Federico II University of Naples, Via Pansini 5, 80131 Naples, Italy

**Keywords:** Diabetes, Coronary lesion, Fractional flow reserve, Hyperemia, Contrast medium, Adenosine, Resting metrics, Instantaneous wave-free ratio

## Abstract

**Background:**

Adenosine-free coronary pressure wire metrics have been proposed to test the functional significance of coronary artery lesions, but it is unexplored whether their diagnostic performance might be altered in patients with diabetes.

**Methods:**

We performed a post-hoc analysis of the CONTRAST study, which prospectively enrolled an international cohort of patients undergoing routine fractional flow reserve (FFR) assessment for standard indications. Paired, repeated measurements of all physiology metrics (Pd/Pa, iFR, contrast-based FFR, and FFR) were made. A central core laboratory analyzed blinded pressure tracings in a standardized fashion.

**Results:**

Of 763 subjects enrolled at 12 international centers, 219 (29%) had diabetes. The two groups were well-balanced for age, clinical presentation (stable or unstable), coronary vessel studied, volume and type of intracoronary contrast, and volume of intracoronary adenosine. A binary threshold of cFFR ≤ 0.83 produced an accuracy superior to both Pd/Pa and iFR when compared with FFR ≤ 0.80 in the absence of significant interaction with diabetes status; indeed, accuracy in subgroups of patients with or without diabetes was similar for cFFR (86.7 vs 85.4% respectively; p = 0.76), iFR (84.2 vs 80.0%, p = 0.29) and Pd/Pa (81.3 vs 78.9%, p = 0.55). There was no significant heterogeneity between patients with or without diabetes in terms of sensitivity and specificity of all metrics. The area under the receiver operating characteristic (ROC) curve was largest for cFFR compared with Pd/Pa and iFR which were equivalent (cFFR 0.961 and 0.928; Pd/Pa 0.916 and 0.870; iFR 0.911 and 0.861 in diabetic and non-diabetic patients respectively).

**Conclusions:**

cFFR provides superior diagnostic performance compared with Pd/Pa or iFR for predicting FFR irrespective of diabetes (clinicaltrials.gov identifier NCT02184117).

## Background

Due to its multi-test validation and cost-effective improvement in clinical outcomes demonstrated by multiple randomized trials as well as observational data, physiological assessment of coronary stenoses by fractional flow reserve (FFR) has emerged as the gold standard for making revascularization decisions on stable lesions [1–5]. Importantly, FFR requires hyperemia, but that does add a small cost and risk [1, 6, 7]. Therefore, the CONTRAST (Can cONTrast Injection Better Approximate FFR compAred to Pure reSTing Physiology?) study recently investigated whether contrast medium could provide an easy alternative and inexpensive tool for assessing FFR (namely contrast FFR, cFFR). It demonstrated that cFFR was superior to resting measurements (rest distal pressure [Pd]/aortic pressure [Pa], and the instantaneous wave-free ratio [iFR]) in terms of diagnostic performance to predict FFR [8].

Diabetes mellitus increases cardiovascular risk, which has been attributed mainly to its detrimental effects on vascular function [9–13]. Before contributing to the development of structural vascular changes or significant coronary artery disease (CAD), diabetes impairs endothelial function leading to microvascular dysfunction [12–18]. Even in diabetic patients without additional cardiac risk factors, endothelial dysfunction has been demonstrated and explained by associated autonomic dysfunction, chronic hyperglycemia, and insulin resistance.

Diabetes does not seem to significantly impact FFR accuracy or its interpretation [4, 19–22], although it can produce coexisting epicardial lesions (quantified by FFR) and microvascular dysfunction (often quantified by measures of hyperemic resistance). Because of potential alterations in microvascular reactivity, cFFR might perform differently in diabetic patients. Thus the aim of this study was to explore whether diabetes might impact the diagnostic ability of cFFR compared with Pd/Pa or iFR versus adenosine-derived FFR ≤ 0.80.

## Methods

### Study population

We explored the impact of diabetes in a post-hoc analysis of the CONTRAST study (clinicaltrials.gov identifier NCT02184117). The design and results of this study have been previously published [8]. Briefly summarized, CONTRAST was an investigator-initiated, prospective diagnostic accuracy study that enrolled a multicenter, international cohort of patients undergoing routine FFR assessment for standard indications. Patients were recruited from 12 centers between June 2014 and April 2015. Subsequent care proceeded as per routine from the clinical FFR measurement without further study-related follow-up. Each subject gave informed consent as approved by the local institutional review board of that participating center. Subjects were excluded in case of prior coronary bypass surgery, known severe cardiomyopathy (LV ejection fraction <30%) or LV hypertrophy (septal wall thickness >13 mm), contraindication to adenosine, or renal insufficiency such that an additional 12–20 ml of contrast would, in the opinion of the operator, pose an unwarranted risk. In cases of multivessel disease, only the first lesion studied using FFR was included. Culprit lesions for an acute infarction were excluded, but non-culprit lesions were permitted. Standard demographic, clinical, and catheterization parameters were collected for each subject.

### Measurements and core lab analysis

The physiology protocol and core lab analysis used for the study has been previously described [8]. Briefly, an initial period of at least 1 min provided a stable assessment of resting physiology without further contrast injection. Next, a manual or injector-based IC bolus of contrast medium was given as per local practice for diagnostic angiography. The volume and type of IC contrast medium were not mandated but varied among sites and even among subjects at a single site but with a strong recommendation for 6–10 ml. A second IC bolus of contrast medium was injected using the same technique when conditions had returned to baseline (after approximately 1 min). Next, after the return of baseline conditions, a manual IC bolus of adenosine was administered (dose at operators’ discretion but with a strong recommendation for 100–200 μg). A second, identical bolus of IC adenosine was given after 1 min.

A subsequent period of at least 1 min provided a second assessment of resting physiology before starting an adenosine infusion at a standard rate of 140 μg/kg/min via either a central or wide bore peripheral IV. The duration of the infusion was approximately 2 min, but could be prolonged or abbreviated as necessary. After stopping IV adenosine and waiting approximately 2 min for conditions to return to baseline, a second, identical IV adenosine infusion was performed. At the end of the procedure, an optional but recommended drift check was performed by bringing the pressure sensor back to the guide catheter at the same location as equalization.

All pressure tracings were sent to the Cardiovascular Research Foundation physiology core lab for standardized and centralized review. The core lab carried out its post-hoc analysis without knowledge of the locally determined Pd/Pa value, IC substance (contrast medium or adenosine), enrolling site, or subject/lesion characteristics.

### Statistical analysis and endpoints

The primary endpoint was accuracy against FFR ≤ 0.80 and compared using a McNemar test between metrics. Secondary endpoints included the area under the receiver operating characteristic (ROC) curve (compared using the DeLong method), sensitivity, and specificity. All the analyses were performed in diabetic and non-diabetic patients. Based on previous evidence [8], the binary thresholds were as follows: FFR ≤ 0.80; cFFR ≤ 0.83; Pd/Pa < 0.92 and iFR < 0.90.

Continuous variables are presented as mean ± standard deviation and were compared with independent samples Student t test. Categorical variables are expressed as count and percentages and were compared with Chi square or Fisher exact tests as appropriate. A Cox proportional model was used for univariate and multivariate analysis to explore the role of diabetes in predicting the cFFR disagreement with FFR. Applicable tests were two-tailed, and p < 0.05 was considered statistically significant. Analyses were performed in SPSS, version 23.0 (SPSS Inc., Chicago, IL, USA) and MedCalc version 12.5 (MedCalc Software, Ostend, Belgium).

## Results

Of 763 subjects, 219 (29%) were diabetic. Compared with non-diabetic patients, those with diabetes were more frequently women, had a higher body mass index, and more frequently had hypertension or dyslipidemia (Table 1). The 2 groups were well-balanced for clinical presentation (stable or unstable), coronary vessel studied, volume and type of intracoronary contrast, and volume of intracoronary adenosine (Table 1).

**Table 1:** Baseline characteristics in patients with or without diabetes

	No diabetes (N = 544)	Diabetes (N = 219)	p value
Age (years)	65.4 ± 9.7	66.4 ± 9.7	0.24
Male	74.8% (407)	63.9% (140)	0.003
BMI (kg/m^2^)	26.8 ± 4.5	28.6 ± 5.0	<0.0001
Smoking (current or past)	47.8% (260)	47.0% (103)	0.85
Hypertension	67.5% (367)	81.3% (178)	<0.0001
Dyslipidemia	61.4% (334)	79.5% (174)	<0.0001
Family history of CAD	26.1% (142)	22.4% (49)	0.28
Renal dysfunction (eGFR < 60 ml/min)	8.6% (47)	12.3% (27)	0.12
Prior MI	24.1% (131)	30.6% (67)	0.06
Prior PCI	14.0% (76)	17.4% (38)	0.24
Peripheral vascular disease	3.7% (20)	6.4% (14)	0.10
Clinical presentation			0.37
Stable	79.2% (431)	76.3% (167)	
ACS	20.8% (113)	23.7% (52)	
Unstable Angina	9.2% (50)	15.5% (34)	
NSTEMI	10.7% (58)	6.8% (15)	
STEMI	0.9% (5)	1.4% (3)	
Coronary vessel			0.86
Left main	3.1% (17)	3.7% (8)	
LAD	60.7% (330)	59.4% (130)	
LCx	17.5% (95)	19.6% (43)	
RCA	18.8% (102)	17.4% (38)	
Contrast medium			0.03
Iobitridol	5.3% (29)	5.0% (11)	
Iodixanol	24.3% (132)	26.0% (57)	
Iohexol	15.8% (86)	9.1% (20)	
Iomeprol	31.1% (169)	26.5% (58)	
Iopamidol	1.3% (7)	0.5% (1)	
Iopromide	7.9% (43)	11.9% (26)	
Ioversol	8.5% (46)	10.0% (22)	
Ioxaglate	5.9% (32)	11.0% (24)	
Volume of IC contrast (ml)	7.9 ± 1.5	7.7 ± 1.6	0.03
5	2.0% (11)	2.7% (6)	
6–7	39.7% (216)	49.3% (108)	
8–9	30.1% (164)	21.0% (46)	
10	27.9% (152)	26.9% (59)	
12	0.2% (1)	0% (0)	
Dose of IC adenosine* (μg)	166.3 ± 46.1	171.2 ± 47.1	0.27
<80	2.3% (9)	1.3% (2)	
80–90	7.1% (28)	7.1% (11)	
100–150	28.4% (112)	29.0% (45)	
160–200	48.5% (191)	45.2% (70)	
>200	13.7% (54)	17.4% (27)	

*ACS* acute coronary syndrome, *BMI* body mass index, *CAD* coronary artery disease, *GFR* glomerular filtration rate, *IC* intracoronary, *LAD* left anterior descending coronary artery, *LCx* left circumflex coronary artery, *MI* myocardial infarction, *NSTEMI* non ST-segment elevation MI, *PCI* percutaneous coronary intervention, *RCA* right coronary artery, *STEMI* ST-segment elevation MI

* Only 549 of 763 patients received IC adenosine, while all other rows are based on 763 total

**Fig. 1:**
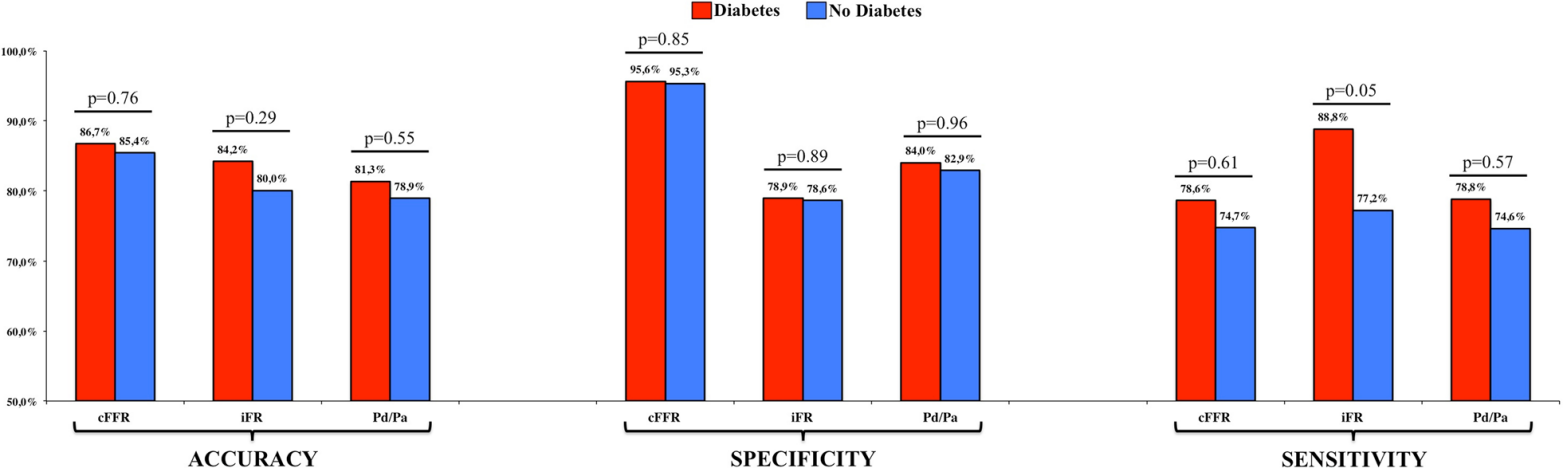
Diagnostic performance of cFFR, iFR and Pd/Pa in patients with or without diabetes. Accuracy, specificity, and sensitivity of each metric are reported for patients with (*red bars*) and without (*blue bars*) diabetes, demonstrating absence of heterogeneity in these subgroups

As reported previously, a binary threshold of cFFR ≤ 0.83 produced an accuracy of 85.8%, superior to both Pd/Pa 78.5% and iFR 79.9% (McNemar p < 0.001 versus both resting metrics), when compared with FFR ≤ 0.8 [8]. In this substudy, there was no significant interaction between diabetes and accuracy; indeed, accuracy in subgroups of patients with or without diabetes was similar for cFFR (86.7 vs 85.4% respectively; p = 0.76), iFR (84.2 vs 80.0% respectively; p = 0.29) and Pd/Pa (81.3 vs 78.9% respectively; p = 0.55) (Fig. 1). Overall, sensitivity was similar among the 3 metrics when comparing non-diabetics and diabetics, respectively (cFFR 78.6 vs 74.7%, p = 0.61; iFR 88.8 vs 77.2%, p = 0.05; Pd/Pa 78.8 vs 74.6%, p = 0.57), but cFFR improved specificity compared with iFR or Pd/Pa and there was no significant interaction with diabetes status (cFFR 95.6 vs 95.3%, p = 0.85; iFR 78.9 vs 78.6%, p = 0.89; Pd/Pa 84.0 vs 82.9%, p = 0.96) (Fig. 1).

**Fig. 2:**
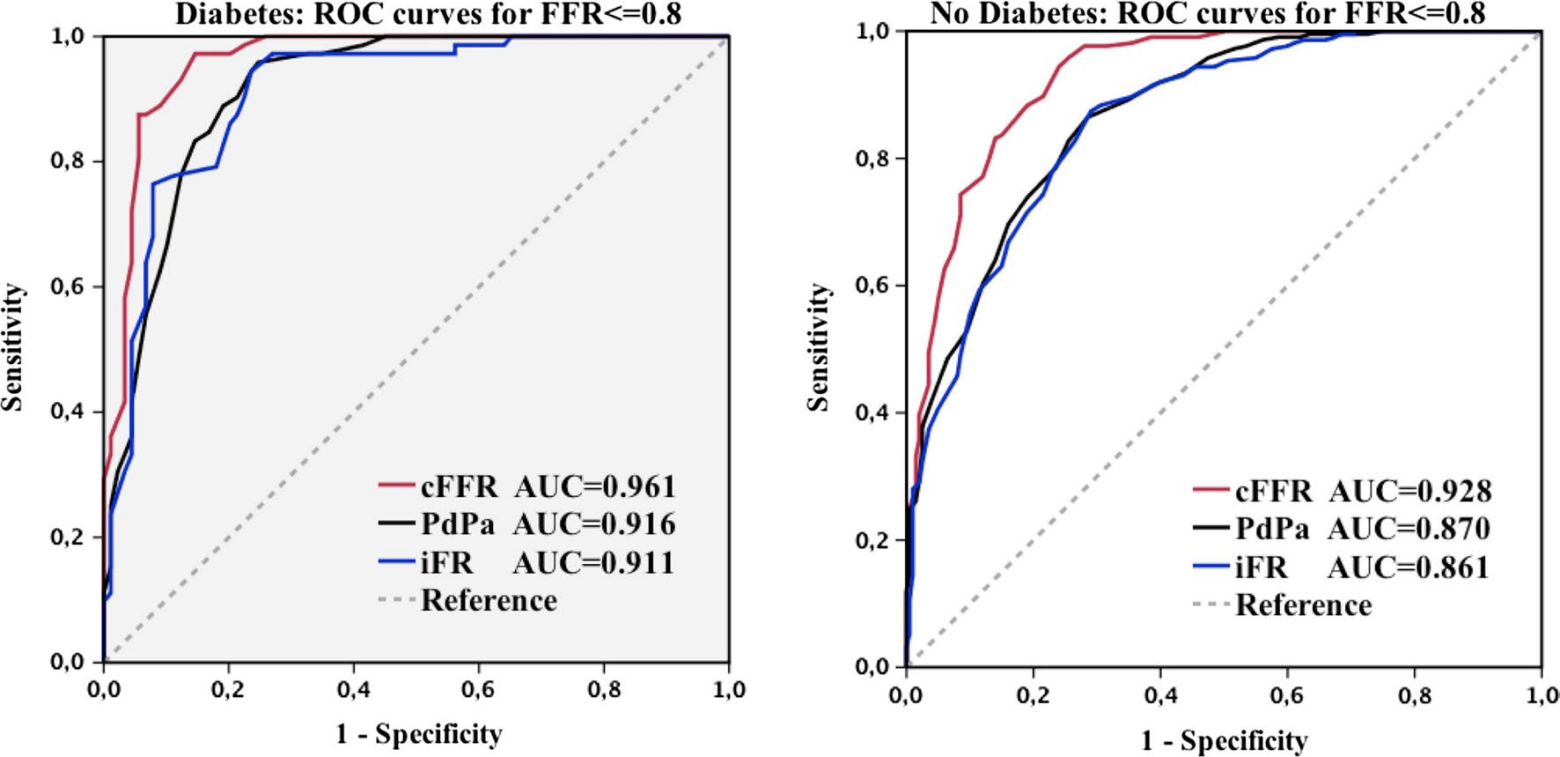
Diagnostic performance expressed by the area under the ROC curve for each metric in patients with or without diabetes. In patients with or without diabetes, contrast FFR (*red line*) has the largest area under the ROC curve, while iFR (*blue line*) and Pd/Pa (*black line*) have equivalent areas

The area under the ROC curve was largest for cFFR compared with Pd/Pa and iFR which were equivalent in both diabetic (0.961 cFFR, 0.916 Pd/Pa, 0.911 iFR; DeLong p = 0.003 for cFFR vs iFR, p < 0.0001 for cFFR vs Pd/Pa and p = 0.66 for iFR vs Pd/Pa; Fig. 2) and non-diabetic patients (0.928 cFFR, 0.870 Pd/Pa, 0.861 iFR; DeLong p < 0.0001 for cFFR vs iFR, p < 0.0001 for cFFR vs Pd/Pa and p = 0.13 for iFR vs Pd/Pa; Fig. 2). There were no significant differences when ROC curves in diabetic and non-diabetic patients were compared (cFFR 0.961 vs 0.928, DeLong p = 0.08; Pd/Pa 0.916 vs 0.870, DeLong p = 0.09; iFR 0.911 vs 0.861, DeLong p = 0.08).

**Fig. 3:**
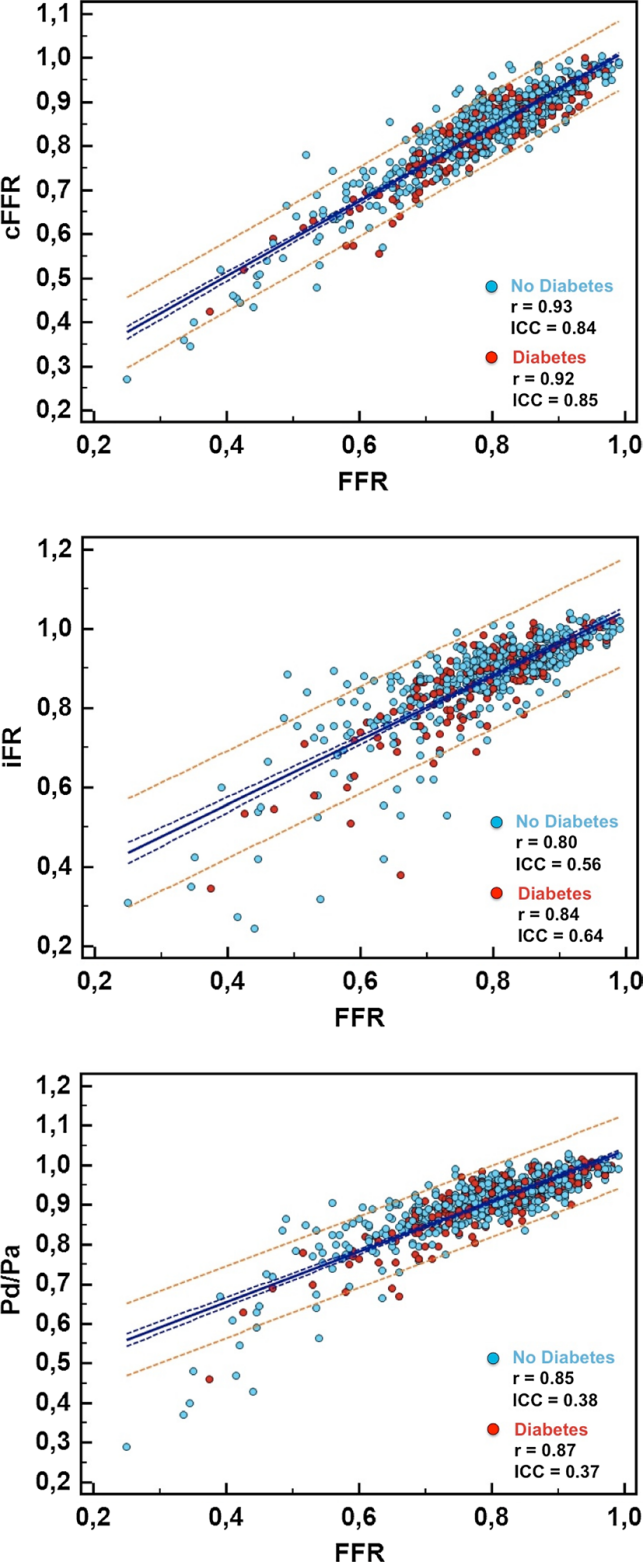
Scatterplots of each metric with FFR in patients with or without diabetes. Resting physiology (either Pd/Pa or iFR) displays a less linear and more scattered relationship with FFR than does modest hyperemia (contrast FFR) in both diabetic (*red dots*) and non-diabetic patients (*blue dots*), as shown visually by the raw data and its local regression (*thick blue line with dashed* 95% confidence intervals) and quantified by correlation coefficients (r from Pearson, ICC from intraclass correlation)

As a physiologic explanation for the superior diagnostic performance of cFFR compared to resting physiology (either Pd/Pa or iFR), Fig. 3 shows that the modest hyperemia induced by cFFR determines a more linear and less scattered relationship versus FFR compared to that of resting physiology, irrespective of diabetes status. Indeed, compared with Pd/Pa and iFR, the correlation of cFFR with FFR was superior in both diabetic (r = 0.92 and ICC = 0.85 for cFFR; r = 0.84 and ICC = 0.64 for iFR; r = 0.87 and ICC = 0.37 for Pd/Pa) or non-diabetic patients (r = 0.93 and ICC = 0.84 for cFFR; r = 0.80 and ICC = 0.56 for iFR; r = 0.85 and ICC = 0.38 for Pd/Pa; Fig. 3).

Diabetes did not predict agreement/disagreement between cFFR and FFR (p = 0.66) in a univariate analysis. Also when diabetes was forced into a multivariable model, it did not significantly influence the accuracy of cFFR (adjusted p = 0.71) (Table 2).

**Table 2:** Predictors of cFFR disagreement with FFR

	Univariate	Multivariate
	p value	p value
Age (years)	0.06	0.34
Male	0.54	
BMI (kg/m^2^)	0.11	0.59
Diabetes	0.66	0.71
Smoking (current or past)	0.59	
Hypertension	0.50	
Dyslipidemia	0.36	
Family history of CAD	0.87	
Renal dysfunction (eGFR <60 ml/min)	0.09	0.30
Prior MI	0.58	
Prior PCI	0.89	
Peripheral vascular disease	0.42	
Clinical presentation (stable or ACS)	0.89	
Coronary vessel	0.045	0.89
Syntax segment	0.004	0.26
Contrast medium	0.004	0.44
Volume of IC contrast	0.005	0.37
Dose of IC adenosine*	0.038	0.76

*ACS* acute coronary syndrome, *BMI* body mass index, *CAD* coronary artery disease, *GFR* glomerular filtration rate, *IC* intracoronary, *MI* myocardial infarction, *PCI* percutaneous coronary intervention

* Only 549 of 763 patients received IC adenosine, while all other rows are based on 763 total

## Discussion

This sub-analysis of the CONTRAST study shows that there is no significant interaction between diabetes and the diagnostic performance of cFFR, therefore, the findings of the main analysis can be extended to patients with diabetes. Contrast FFR provides superior diagnostic performance compared with resting metrics (iFR and Pd/Pa) irrespective of diabetic status.

The CONTRAST study is the largest study to investigate the diagnostic performance of cFFR and the present sub-analysis is the first to explore the impact of diabetes on cFFR performance compared with resting methods [23–25].

Diabetes mellitus is a well-known risk factor for cardiovascular disease [9–11]. The presence of diabetes accelerates the natural course of atherosclerosis, involving both proximal epicardial and distal small vessel coronary arteries with greater plaque ulceration and thrombosis and microvascular disease. Patients with diabetes have a variety of structural and functional microvascular coronary abnormalities [12–18] that may translate into a reduction of hyperemic blood flow due to an impairment of microcirculatory vasodilation.

Some studies investigated the accuracy of FFR in diabetic patients. Yanagisawa et al. compared FFR performance in 96 and 149 patients with or without diabetes respectively and observed that there was no significant difference, although within diabetic patients, those with a poor glycemic control (glycated hemoglobin >7%) had a lower FFR specificity [19]. Domínguez-Franco et al. found that deferring PCI in diabetic patients (29% of all patients) with a moderately severe coronary artery stenosis based on the FFR value was as safe as in patients without diabetes [20]. Sahinarslan et al. analyzed 122 patients (29.5% with diabetes) with intermediate coronary lesions and observed that there was no difference between the FFR values in patients with or without diabetes who had been paired according to reference vessel diameter and degree of luminal narrowing of coronary lesions [21]. Reith et al. evaluated FFR in 266 intermediate grade lesions of 224 patients (113 non-diabetics and 111 diabetics) with stable CAD, also exploring the role of adequate glycemic control (defined as glycated hemoglobin <7%). They found that FFR accuracy was not affected by diabetic status and its glycemic control [22]. In the FAME-2 trial, the FFR guided PCI was superior to medical therapy for the primary end point (composite of death from any cause, nonfatal myocardial infarction, or urgent revascularization within 2 years) in patients with or without diabetes (28 and 72% respectively) with an absence of significant interaction between the two sub-groups interaction p = 0.50) [4].

Interestingly, some data have questioned the accuracy of FFR is some cases. Although FFR and coronary flow velocity reserve (CFVR) have an equivalent diagnostic accuracy for inducible myocardial ischemia, it has been shown that they provide discordant results in 30–40% of cases, which was suggested to have a significant impact on clinical outcomes, particularly when FFR is normal and CFVR abnormal that generally indicates predominant microvascular involvement in coronary artery disease [26, 27]. Therefore it cannot be excluded that the accuracy of FFR, and thus also of cFFR, may be affected in case of microvascular disease as occurs in patients with diabetes. Indeed, a recent study has showed that in patients with diabetes, particularly those with previous MI, FFR-based deferred revascularization was associated with poor medium-term outcomes suggesting that the combining of FFR with imaging techniques may be useful to guide our treatment strategy in these patients with high-risk, fast-progressing disease [28]. Comparing FFR-guided deferred revascularization in patients with and without diabetes, Kennedy et al. also showed that those with diabetes were associated with a significantly higher rate of target lesion failure [29]. Accordingly, a recent study by Liu et al. showed that a higher FFR was associated with lower rates of death, myocardial infarction and revascularization among non-diabetic patients with deferred PCI, but in diabetic patients with deferred revascularization, FFR was not able to differentiate the risk of cardiovascular events [30].

Whether intravascular imaging, may impact on this higher failure rate remains unexplored, but the COMBINE study, the first prospective multicenter combining FFR and optical coherence tomography, will contribute to clarify this issue investigating the hypothesis that adding the study of plaque morphology to FFR in intermediate lesions may better predict events in patients with diabetes [31].

Although few data exist on the clinical value of FFR in diabetic patients, the impact of diabetes and any potentially associated microvascular dysfunction was previously unknown for cFFR performance. Indeed, whether the submaximal vasodilatation induced by contrast medium compared with adenosine might affect the accuracy of cFFR measurement in patients with diabetes had never been explored before. Our novel results found no significant heterogeneity between subgroups stratified by diabetes, and that the overall results of the CONTRAST study can be extended to all patients irrespective of diabetic status. Therefore, cFFR has been proven accurate and superior to resting metrics in patients with diabetes as well as in those without diabetes.

### Limitations

This is an unplanned post-hoc analysis of the CONTRAST study, which was not designed or powered to investigate the diabetic subgroup specifically. Therefore, our exploratory analysis should be considered hypothesis-generating and needs to be confirmed in further trials. Additionally, our sub-analysis shares the limitations of the main study [8], namely the short-acting nature of contrast-induced hyperemia and lack of clinical data regarding contrast-induced nephropathy. Finally, being cFFR measured during submaximal hyperemia, it shares the same FFR limitations related to hyperemia in the setting of microvascular dysfunction.

## Clinical relevance and conclusions

When performing physiological lesion assessment, some operators prefer to avoid adenosine due to rare side effects and minimal but nonzero added time and expense. Moreover, adenosine is still expensive or unavailable in some areas of the world, and sporadic patients have contraindications.

Contrast FFR provides superior diagnostic performance than Pd/Pa or iFR for predicting FFR irrespective of diabetes. Although FFR remains the reference standard for diagnostic certainty, for clinical scenarios or healthcare systems in which adenosine is contraindicated or prohibitively expensive, cFFR may represent a simple alternative technique to adenosine-FFR for invasive coronary physiological assessment in diabetic as well as non-diabetic patients.

## Abbreviations

CAD: coronary artery disease
cFFR: contrast-based fractional flow reserve
ECG: electrocardiogram
FFR: fractional flow reserve
IC: intracoronary
iFR: instantaneous wave-free ratio
IQR: interquartile range
IV: intravenous(ly)
Pd/Pa: resting ratio of distal coronary pressure to aortic pressure
ROC: receiver operating characteristic curve
